# Proceedings of the Central Ohio Dental Association

**Published:** 1868-07

**Authors:** 


### PROCEEDINGS OF THE CENTRAL OHIO DENTAL ASSOCIATION.

The Third Annual Meeting of the Central Ohio Dental Association was held in the Philharmonic Hall, of the city of Mansfield, on Tuesday and Wednesday, May 12th and 13th. The meeting was called to order at 2 o'clock P. M., by the President, Dr. M. DeCamp, of Mansfield.

Many subjects of interest to the profession were discussed. One of the principal subjects considered was the enforcement of the code of ethics adopted by the Association. During this discussion it was the expressed desire and determination of all present, to maintain the integrity of true professional character in the Association, and by maintaining this standard, make membership an honor.

On motion several members were expelled for neglect of professional duties. Written charges were then preferred against Drs. C. M. Kelsey and W. F. Semple, of Mt. Vernon, also against Drs. Beauman and Dunn, of Columbus, for unprofessional conduct. They were notified to appear at the next meeting of the Association to answer said charges.

Essays were read by Drs. R. G. Warner and G. W. DeCamp.

Officers elected for ensuing year were:

*President.*—Dr. Harrington, of Newark.

*Vice-President.*—D. E. Chidester, of Massilon.

*Recording Secretary.*—Dr. G. W. DeCamp, of Mansfield.

*Corresponding Secretary.*—Dr. J. R. Jennings, of Ravenna.

*Treasurer.*—Dr. S. B. Stewart, of Akron.

The meeting was very pleasant and profitable to all. The attendance was good, most of the prominent Dentists of Central Ohio being present.

The Association adjourned on Wednesday evening, at 11 o'clock, to meet in Newark, on the second Tuesday of July, 1869.

